# Col1α2-Cre-mediated recombination occurs in various cell types due to Cre expression in epiblasts

**DOI:** 10.1038/s41598-023-50053-z

**Published:** 2023-12-18

**Authors:** Yuzuru Matsumoto, Shinya Ikeda, Takeshi Kimura, Koh Ono, Noboru Ashida

**Affiliations:** 1 Department of Cardiovascular Medicine, Graduate School of Medicine, Kyoto University, 54 Kawahara-cho, Shogoin, Sakyo-ku, Kyoto 606-8507 Japan; 2 Present Address: Department of Pharmacology, Shiga University of Medical Science, Shiga, Japan; 3 Present Address: Hirakata Kohsai Hospital, Osaka, Japan; 4 Present Address: College of Pharmaceutical Sciences, Ritsumeikan University, Shiga, Japan

**Keywords:** DNA recombination, Genetic engineering

## Abstract

The Cre-LoxP system has been commonly used for cell-specific genetic manipulation. However, many Cre strains exhibit excision activity in unexpected cell types or tissues. Therefore, it is important to identify the cell types in which recombination takes place. Fibroblasts are a cell type that is inadequately defined due to a lack of specific markers to detect the entire cell lineage. Here, we investigated the Cre recombination induced by Col1α2-iCre, one of the most common fibroblast-mesenchymal Cre driver lines, by using a double-fluorescent Cre reporter line in which GFP is expressed when recombination occurs. Our results indicated that Col1α2-iCre activity was more extensive across cell types than previously reported: Col1α2-iCre-mediated recombination was found in not only cells of mesenchymal origin but also those of other lineages, including haematopoietic cells, myocardial cells, lung and intestinal epithelial cells, and neural cells. In addition, study of embryos revealed that recombination by Col1α2-iCre was observed in the early developmental stage before gastrulation in epiblasts, which would account for the recombination across various cell types in adult mice. These results offer more insights into the activity of Col1α2-iCre and suggest that experimental results obtained using Col1α2-iCre should be carefully interpreted.

## Introduction

The Cre-LoxP system has been a commonly used method for conditional genetic manipulations. However, it has been noted that the majority of Cre strains exhibit some degree of unintended recombination^1^. Since the distribution and intensity of Cre recombinase activity have impacts on the interpretation of experiments, investigators must consider how such unintended recombination might influence their results^2^.

Fibroblasts are found in connective tissue throughout the body and play an important role not only in wound healing but also in development, homeostasis, ageing and disease^3^. The majority of fibroblasts derives from the precursors of paraxial mesoderm and lateral plate mesoderm, whereas multiple cell lineages converge to form fibroblasts, for example cardiac fibroblasts are generated from epicardial and endocardial epithelial cells through epithelial-to-mesenchymal transition (EMT) and endothelial-to-mesenchymal transition (EndMT) respectively^4^. So, they are recognized as one of the most difficult cell types to identify with only one specific marker, and characterized by their morphology and the absence of leucocyte, epithelial and vascular lineage markers^5^. Although many papers have used a variety of Cre-LoxP systems to target fibroblasts, some of them have been reported to express Cre recombinase in not only fibroblasts but also other extracellular-matrix-producing cells and even in immune cells, epithelial cells, and neurons^6^. The collagen gene is commonly expressed by fibroblasts, and the alpha-2 subunit of the fibril-forming type I collagen (Col1α2) has been used as a marker of fibroblasts. A previous report indicated that Cre recombinase was expressed predominantly in cells of mesenchymal origin in noninducible Col1α2-Cre strain *Col1α2-iCre*^7^. However, another paper showed recombination by Col1α2-iCre was observed in cells other than those of mesenchymal origin^8^. We designed this study to solve this inconsistency.

In the report introducing Col1α2-iCre, Rosa26R-lacZ was used as a reporter line and β-galactosidase activity of Col1α2-iCre/Rosa26R-lacZ mice was restricted in the dermis, fibrous connective tissues surrounding internal organs, mesenchymal cells of blood vessel walls and microglial cells in brain^7^. Although Rosa26R-lacZ line has been often used, detection of β-galactosidase enzymatic activity depends on condition of protein, so that over- or underfixation can result in the underrepresentation of Cre recombinase activity. Indeed, several reports have suggested that Rosa26R-lacZ indicator line is less sensitive than some fluorescent indicator lines^9^. In this study, we examined the Cre recombination by Col1α2-iCre, with a double-fluorescent Cre reporter line^9^.

## Results

### Reporter line for detection of Col1α2-iCre characterization

To evaluate the activity of Col1α2-iCre, it was needed to choose the most suitable reporter line. Most Cre reporter lines, in which a marker gene (e.g. lacZ, GFP, CFP, or YFP) is expressed, can label only recombined cells. However, it is desirable to label not only recombined cells but also non-recombined cells for precise evaluation. A dual fluorescent indicator line *Rosa26R-mTmG*, in which mT/mG system expresses membrane-targeted tdTomato (‘‘mT’’) prior to Cre excision and membrane-targeted EGFP (‘‘mG’’) following Cre excision, enables us to distinguish between recombined and non-recombined cells at single-cell resolution^9^. Therefore, Col1α2-iCre mice were bred with Rosa26R-mTmG mice to make Col1α2-iCre/mTmG mice.

### Recombination by Col1α2-iCre was observed in the dermis and epidermis

**Figure 1:**
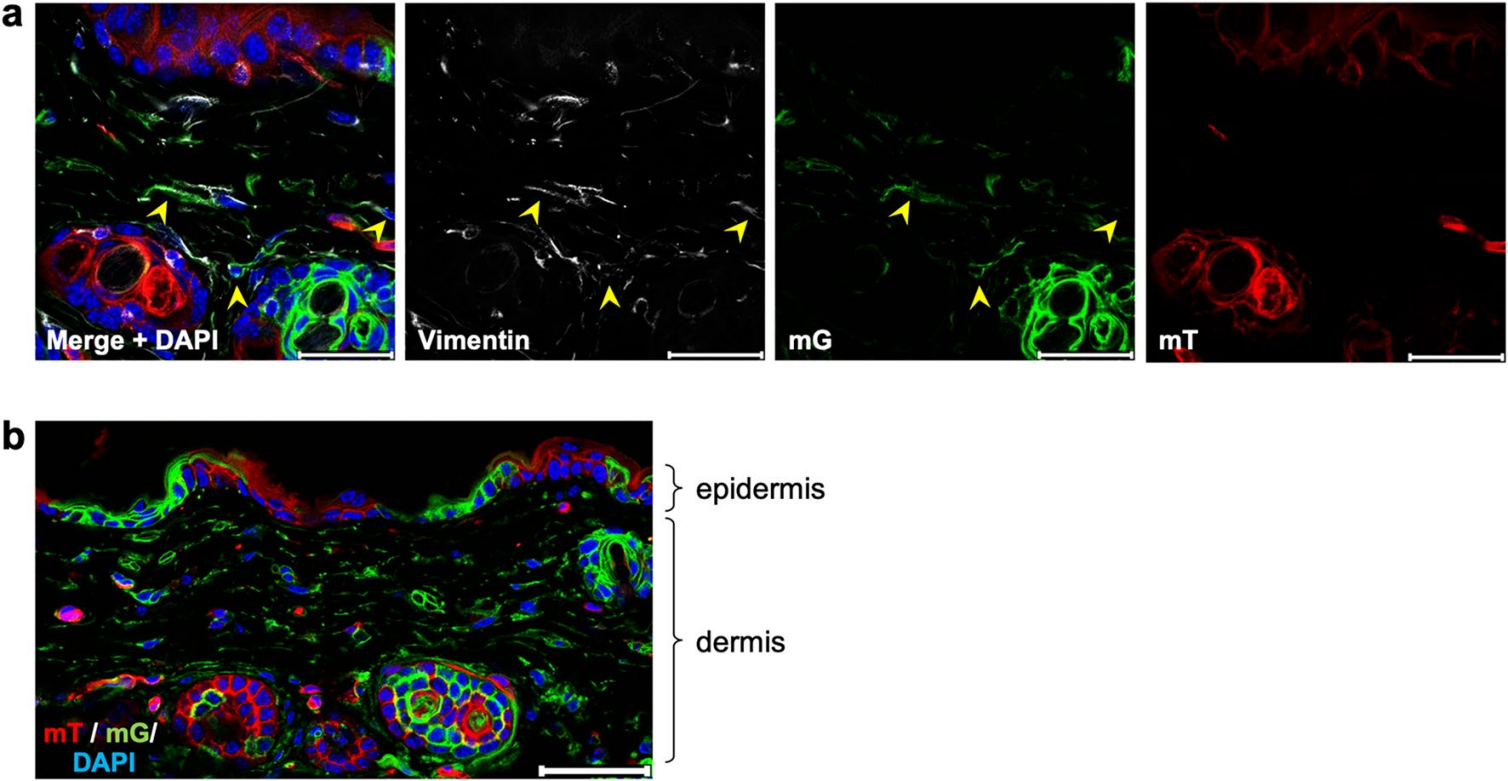
Recombination by Col1α2-iCre in the dermis and epidermis. (**a**) Immunostaining of dermis for vimentin (n = 3). Arrowheads indicate examples of both vimentin- and GFP-positive cells. Scale bar: 30 μm. (**b**) Immunofluorescence image including epidermis (n = 3). Scale bar: 50 μm.

First, we examined the skin of Col1α2-iCre/mTmG mice. Immunofluorescence analysis showed that most fibroblasts (Vimentin +) in the dermis expressed GFP (Fig. 1a), consistent with a previous report^7^. However, another report suggested that recombination by Col1α2-iCre was also observed in the epidermis^8^. We found GFP-positive cells in the epidermis and hair follicles (Fig. 1b). This result indicated extensive Cre activity in cells other than those of mesenchymal origin.

### Recombination by Col1α2-iCre was observed in haematopoietic cells

**Figure 2:**
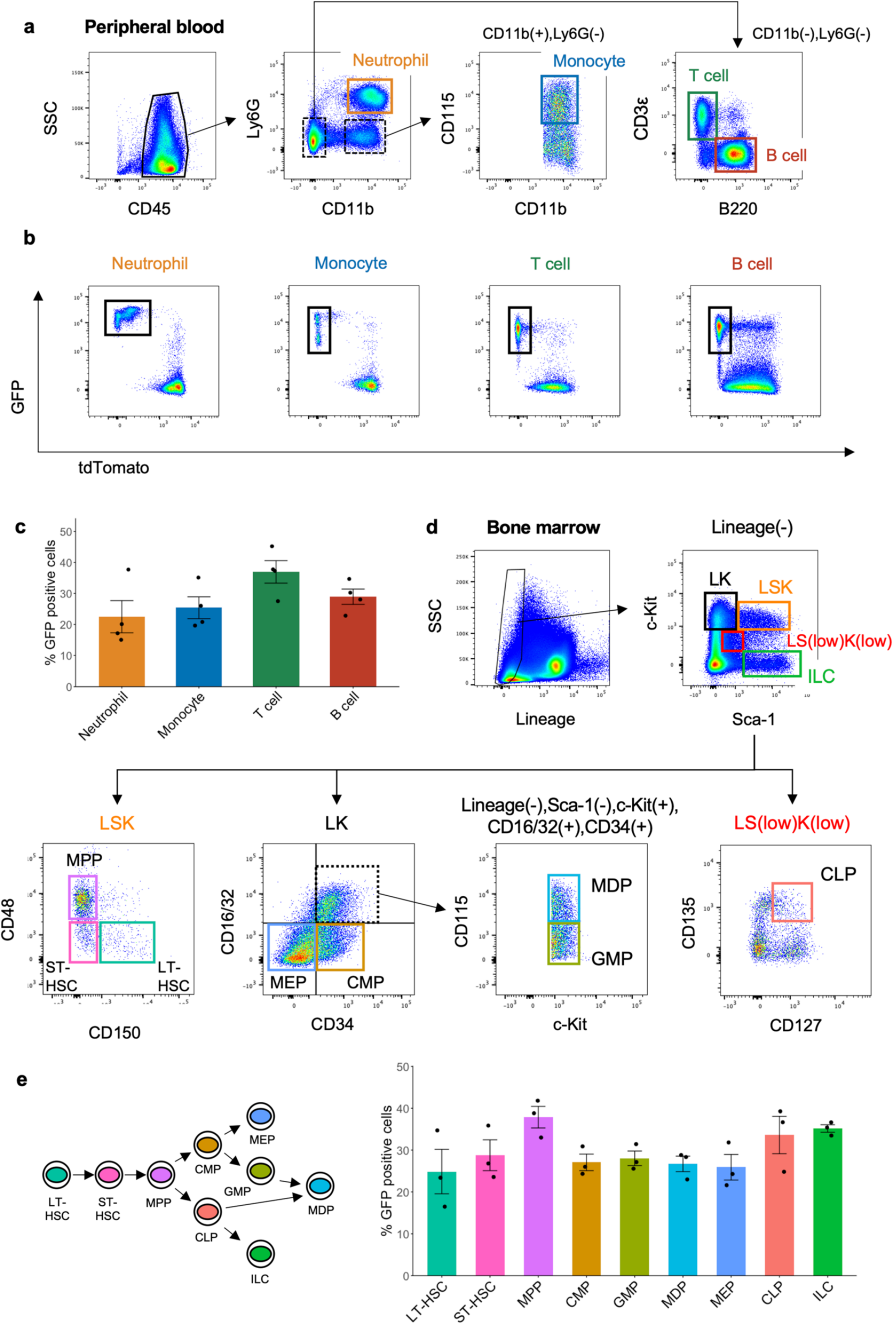
Recombination by Col1α2-iCre in haematopoietic cells. (**a**) Flow cytometry of the peripheral blood cells from Col1α2-iCre/mTmG mice. (**b**) GFP expression in each cell type in the peripheral blood. (**c**) Summary of GFP expression in each cell type in the peripheral blood (n = 4). (**d**) The representative gating schema of bone marrow of Col1α2-iCre/mTmG mice. *LT-HSC* long-term haematopoietic stem cell, *ST-HSC* short-term haematopoietic stem cell, *MPP* multipotent progenitor, *CMP* common myeloid progenitor, *GMP* granulocyte macrophage progenitor, monocyte-macrophage dendritic cell progenitor, *MEP* megakaryocyte-erythrocyte progenitors, *CLP* common lymphoid progenitor, *ILC* innate lymphoid cell. (**e**) Summary of GFP expression in the bone marrow (n = 3).

Next, we investigated recombination in the haematopoietic cells of Col1α2-iCre/mTmG mice because gene manipulation by Col1α2-iCre was reported to also occur in circulating lymphocytes and neutrophil granulocytes^8^. A flow cytometric analysis of peripheral blood showed that there was a GFP-positive population in neutrophils (22.5 ± 5.2%), monocytes (25.4 ± 3.5%), T cells (37.0 ± 3.6%), and B cells (28.9 ± 2.5%) (Fig. 2a–c, Supplementary Table S1). Since Col1α2-iCre-mediated recombination was observed in both myeloid and lymphoid cells, we evaluated the proportion of GFP-positive bone marrow cells. Flow cytometric analysis revealed that GFP-positive cells were observed in long-term haematopoietic stem cells (LT-HSCs; Lineage-, c-Kit + , Sca-1 + , CD150 + , CD48-) (24.9 ± 5.3%), which are the most immature cells in the bone marrow (Fig. 2d,e and Supplementary Fig. S1a, Supplementary Table S1)^10^. Additionally, there were GFP-positive populations in short-term HSCs (ST-HSCs; Lineage −, c-Kit + , Sca1 + , CD150 −, CD48 −) (28.8 ± 3.7%), multipotent progenitors (MPPs; Lineage −, c-Kit + , Sca1 + , CD150-, CD48 +) (37.9 ± 2.6%), downstream myeloid lineages, such as common myeloid progenitors (CMPs; Lineage −, c-Kit + , Sca-1 −, CD34 + , CD16/32 −) (27.1 ± 2.0%), granulocyte macrophage progenitors (GMPs; Lineage −, Sca-1 −, c-Kit + , CD34 + , CD16/32 + , CD115 −) (28.0 ± 1.7%), monocyte-macrophage dendritic cell progenitors (MDPs; Lineage-, Sca-1 −, c-Kit + , CD34 + , CD16/32 + , CD115 +) (26.7 ± 1.9%), and megakaryocyte-erythrocyte progenitors (MEPs; Lineage-, Sca-1 −, c-Kit + , CD34 −, CD16/32-) (25.9 ± 3.1%)^11^, and the lymphoid lineages, including common lymphoid progenitors (CLPs; Lineage-, Sca-1 low, c-Kit low, CD135 + , CD127 +) (33.6 ± 4.5%), innate lymphoid cells (ILCs; Lineage −, Sca-1 + , c-Kit −) (35.2 ± 0.9%)^12^. To confirm recombination in HSCs, we transplanted bone marrow cells from Col1α2-iCre/mTmG mice into wild-type recipients (Supplementary Fig. S1b) and analysed peripheral blood cells from the recipients 4 weeks later. There were GFP-positive cells among neutrophils (39.4 ± 0.5%), monocytes (39.4 ± 0.5%), T cells (26.5 ± 3.8%), and B cells (47.6 ± 3.2%) (Supplementary Fig. S1c), and this result verified that Col1α2-iCre recombination took place in HSCs.

### Cre-mediated recombination was observed in the hearts of Col1α2-iCre/mTmG mice

**Figure 3:**
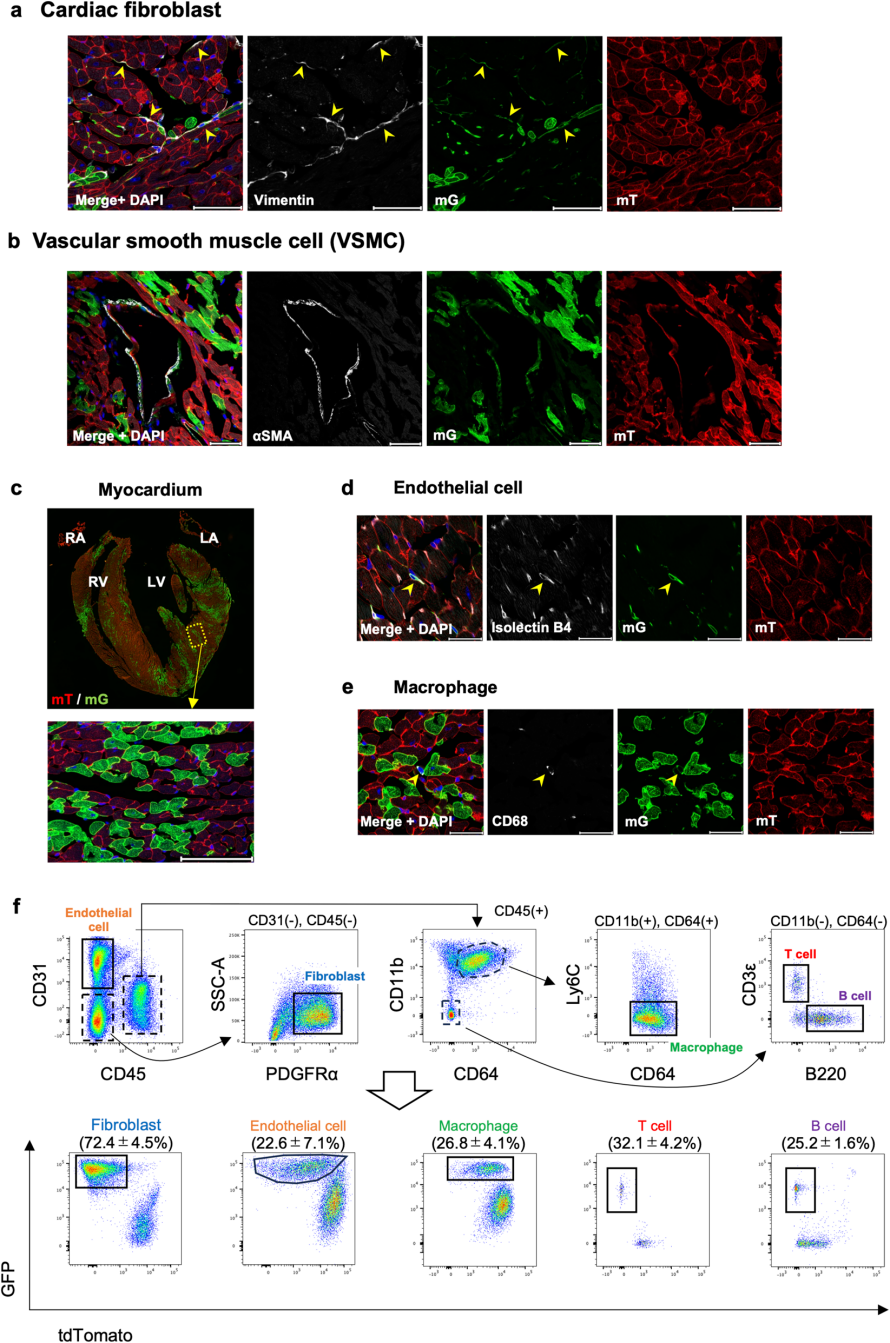
Recombination by Col1α2-iCre across cell types in the heart. (**a**) Immunofluorescence image of cardiac fibroblasts (n = 5). The arrowheads indicate examples of both vimentin- and GFP-positive cells. Scale bar: 50 μm. (**b**) Immunofluorescence image of vascular smooth muscle cells (VSMCs) (n = 5). Scale bar: 50 μm. (**c**) GFP-positive myocardial cells. The upper image is a whole image, and the lower image is a magnified image (n = 5). (**d**) GFP-positive endothelial cell (arrowheads) (n = 5). Scale bar: 50 μm. (**e**) GFP-positive macrophage (arrowheads) (n = 5). Scale bar: 30 μm. (**f**) The representative gating schema of nonmyocytes of Col1α2-iCre/mTmG hearts and GFP expression in each cell type.

Col1α2 has been commonly used as a marker of cardiac fibroblasts^12,13^. However, the cardiac cell types that recombination by Col1α2-iCre takes place in have not been fully elucidated. We evaluated Cre-mediated recombination in the hearts of Col1α2-iCre/mTmG mice by immunohistochemistry and flow cytometric analysis. Histological staining showed that there were many fibroblasts (Vimentin +)^14^ positive for GFP (Fig. 3a), similar to vascular smooth muscle cells (αSMA +) (Fig. 3b), which was consistent with a previous study^7^. Surprisingly, GFP-positive cardiomyocytes (identified by morphology), endothelial cells (Isolectin B4 +)^15^ and tissue macrophages (CD68 +)^16^ were found in the hearts of Col1α2-iCre/mTmG mice (Fig. 3c–e). We further measured the percentage of GFP-positive nonmyocytes with flow cytometry. While 72.4 ± 4.5% of fibroblasts (CD45-, CD31-, PDGFRα +) were positive for GFP, 22.6 ± 7.1% of endothelial cells (CD45-, CD31 +), 26.8 ± 4.1% of tissue macrophages (CD45 + , CD11b + , CD64 + , Ly6C low), 32.1 ± 4.2% of T cells (CD45 + , CD11b-, CD3ε +), and 25.2 ± 1.6% of B cells (CD45 + , CD11b-, B220 +) were also GFP-positive (Fig. 3f, Supplementary Table S1).

### Col1α2-iCre-mediated recombination was widespread in various organs

Considering the results above, Col1α2-iCre-mediated recombination was observed in not only cells of the fibroblast-mesenchymal lineage but also cells of other lineages, such as haematopoietic cells in bone marrow, cardiomyocytes, endothelial cells and immune cells in the heart. We further investigated Col1α2-iCre-mediated recombination in other organs. Histological analysis revealed that GFP-positive cells were observed in myocytes in skeletal muscles, tubular epithelial cells in the kidney, hepatocytes in the liver, pneumocytes in the lung, glandular epithelium in the intestine, and neural cells in the cerebral cortex and retina (Supplementary Fig. S2a–l).

### Col1α2-iCre-mediated recombination occurred in epiblasts

**Figure 4:**
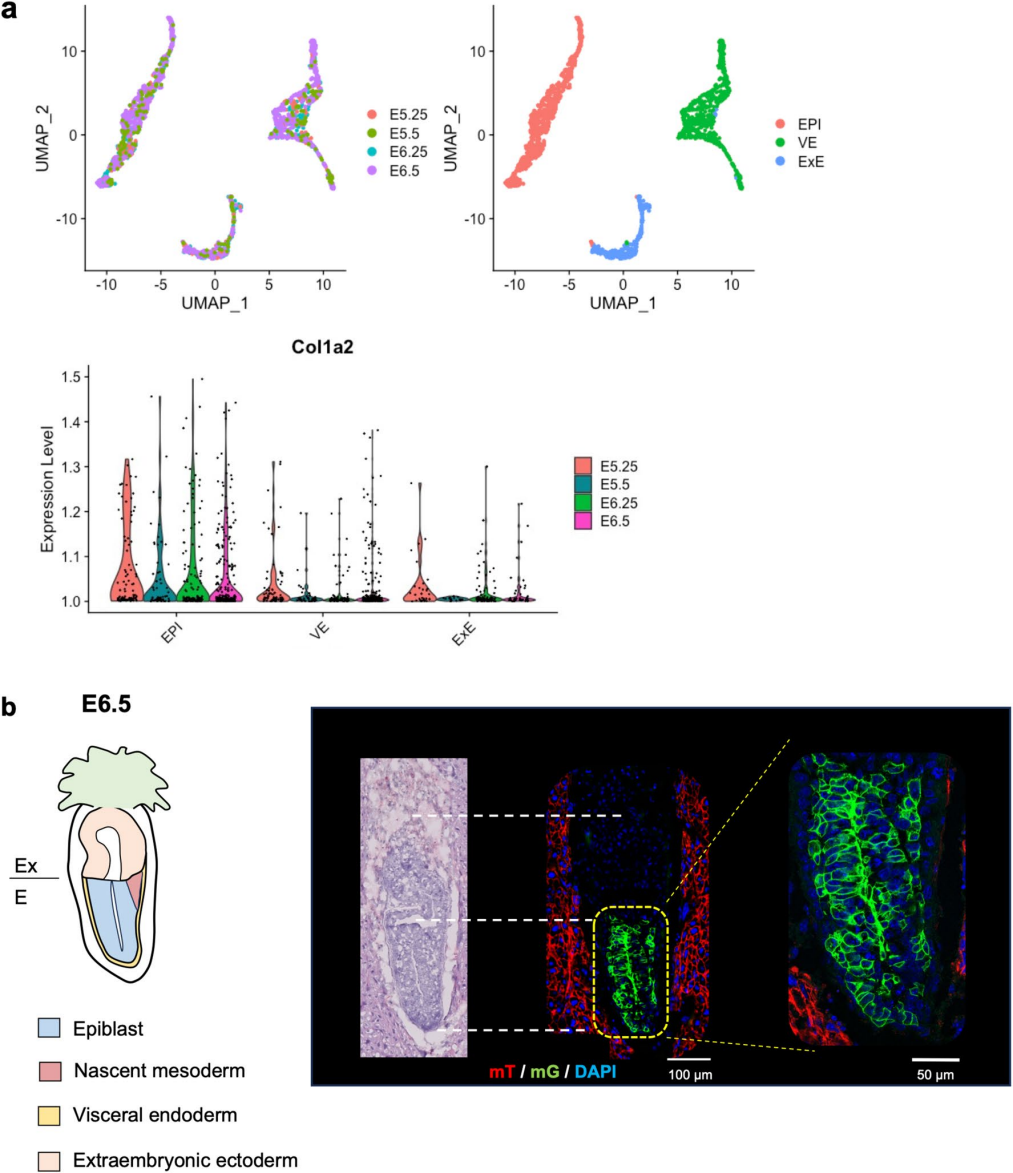
Col1α2-iCre recombination occurred in epiblasts. (**a**) UMAP visualizations of all the cells from E5.25, E5.5, E6.25 and E6.5 together (n = 1724) categorized by embryonic day (top left) and cell type (top right). The lower panel is a violin plot of the Col1α2 gene expression. (**b**) Left: illustration of an E6.5 embryo. Right: HE staining and immunofluorescence images of E6.5 embryos (n = 3). The signal intensity of Tomato in epiblasts was much weaker than that in maternal cells. *EPI* epiblast, *VE* visceral endoderm, *ExE* extraembryonic ectoderm, *Ex* extraembryonic, *E* embryonic.

According to previous studies, the first expression of the endogenous collagen I gene was reported to start at embryonic day (E) 8.5^17,18^, which is the postgastrulation stage. However, it cannot explain the extent of GFP-positive cell types in Col1α2-iCre /mTmG mice. Although the majority of fibroblasts originate from paraxial mesoderm and lateral plate mesoderm^4^, epidermal cells and neural cells are derived from ectoderm; hepatocytes, gut and lung epithelium are from endoderm; cardiomyocytes and intraembryonic haematopoietic cells also come from intraembryonic mesoderm, but they are transcriptomically different from mesenchyme (Supplementary Fig. S3a)^19^. Therefore, our results implicated that Col1α2-iCre expression had started earlier than the three germ layers arise. So, we investigated the timing of endogenous Col1α2 gene expression during mouse embryogenesis by analysing public single-cell RNA sequencing (scRNA-seq) datasets^19,20^. First, we reanalyzed the scRNA-seq dataset of E4.5, E5.25, E5.5, E6.25 and E6.5 murine embryos^20^. Although the Col1α2 gene was not detected in blastocytes at E4.5, Col1α2 mRNA was expressed in the epiblast (EPI) cluster from E5.25 to E6.5 and less in the extraembryonic ectoderm (ExE) and visceral endoderm (VE) cluster (Fig. 4a). Next, we used scRNA-seq datasets spanning from E6.75 to E13.5^19^. They showed that Col1α2 mRNA was expressed predominantly in the allantois, the amniochorionic mesoderm, the splanchnic mesoderm (except for cardiomyocytes), mesenchymal stromal cells, chondrocytes and osteoblast precursors (Supplementary Fig. S3b–f). To validate Col1α2-iCre-mediated recombination before gastrulation, we examined Col1α2-iCre/mTmG mouse embryos at E6.5 by histological analysis and revealed that GFP expression was observed in more than half of the epiblasts and nascent mesoderm, partly in the visceral endoderm, and rarely in extraembryonic lesions (Fig. 4b). This result was fairly consistent with our scRNA-seq analysis, and we concluded that recombination by Col1α2-iCre occurred in epiblasts. Finally, we examined embryonic bodies at E9.5, E13.5 and E17.5 to validate the recombination across cell lineages. GFP-positive cells were found in not only cells of the fibroblast-mesenchymal lineage but also cells of the myocardium, vessel wall, lung, liver, gut, urinary system, nerve system and genital organs at each time point (Supplementary Fig. S4a–c). This widespread recombination was a result of recombination events in epiblasts.

### Col1α2-CreER recombination in skin and heart.

**Figure 5:**
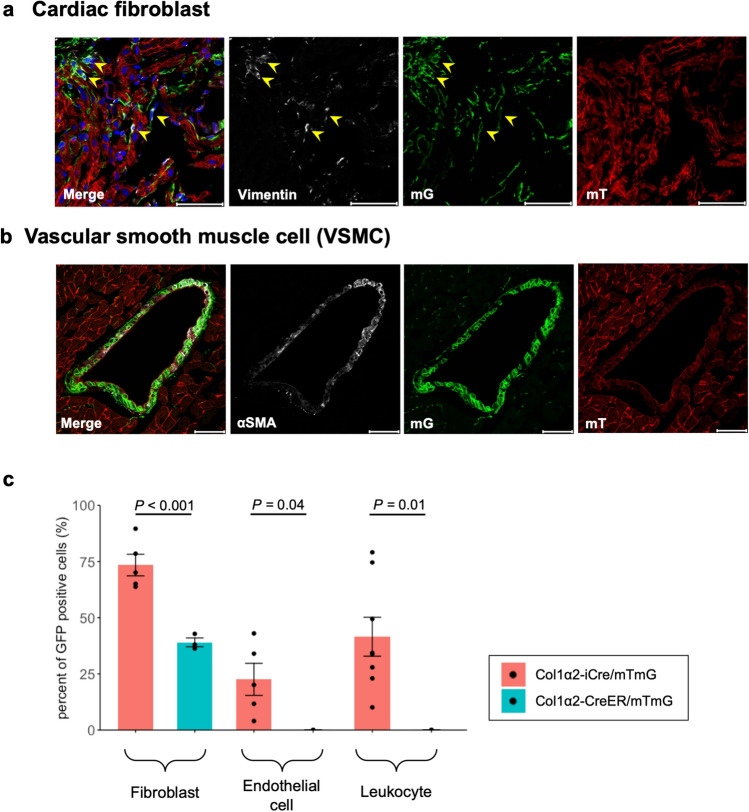
Cre recombination in skin and heart of Col1α2-CreER/mTmG mice. (**a**) Immunofluorescence image of cardiac fibroblasts. Arrowheads indicate examples of both vimentin and GFP-positive cells (n = 3). Scale bar: 50 μm. (**b**) Immunofluorescence image of vascular smooth muscle cells (VSMCs) (n = 3). Scale bar: 50 μm. (**c**) Summary of GFP expression in Col1α2-iCre/mTmG and Col1α2-CreER/mTmG hearts (fibroblast and endothelial cell of Col1α2-iCre/mTmG heart: n = 5, leukocyte of Col1α2-iCre/mTmG heart: n = 8, fibroblast, endothelial cell and leukocyte of Col1α2-CreER/mTmG: n = 3). Statistical significance was analyzed by Student’s t test (fibroblast) and by Wilcoxon rank sum test (endothelial cell and leukocyte).

Considering the popularity of drug-inducible Cre strain, we analyzed an inducible Col1α2-Cre line (Col1α2-CreER)^21^. GFP-positive population was detected in dermal connective tissue, but GFP-positive cells were not detected in epidermis of Col1α2-CreER/mTmG mice (Supplementary Fig. S5a). In the heart of Col1α2-CreER/mTmG mice, GFP-positive cells were found only in fibroblasts (Fig. 5a, Supplementary Fig. S5c) and VSMCs (Fig. 5b), not in cardiomyocytes, endothelial cells and leukocytes (Supplementary Fig. S5b,c). However, comparing to Col1α2-iCre/mTmG hearts, Col1α2-CreER/mTmG hearts showed a significantly reduced recombination efficiency in fibroblasts (39.0 ± 2.0% versus 72.4 ± 4.5%; *P* < 0.001), as well as endothelial cells (0.0 ± 0.0% versus 22.6 ± 7.1%; *P* = 0.04) and leukocytes (0.0 ± 0.0% versus 22.6 ± 7.1%; *P* = 0.01) (Fig. 5c, Supplementary Table S1).

## Discussion

The Cre-LoxP system is widely used for conditional gene targeting. However, many Cre strains, including fibroblast-targeting Cre, have been reported to exhibit some degree of unexpected recombination^1^. In the present study, a double-fluorescent Cre reporter line Rosa26R-mTmG mice, instead of Rosa26R-lacZ mice, demonstrated Col1α2-iCre-mediated recombination in cells other than those of mesenchymal origin. Next, we confirmed that Col1α2-iCre-mediated recombination took place in epiblasts before gastrulation.

First, histological and flow cytometric analyses of adult and prenatal Col1α2-iCre/mTmG mice revealed that Cre-mediated recombination was present in not only cells of mesenchymal origin but also cells of other lineages, including but also cells of other lineages, such as hematopoietic, myocardial, lung, and intestinal epithelial, and neural cells. However, a previous paper, in which Cre recombinase activity of Col1α2-iCre mice was examined by using Rosa26R-lacZ indicator line, reported that β-galactosidase expression was restricted in cells of mesenchymal origin^7^. This inconsistency was attributed to reporter line: Rosa26R-mTmG line might be more sensitive than Rosa26R-lacZ line and the mTmG system allowed us to identify recombined cells thanks to dual membrane-targeted fluorescence.

Second, Col1α2-iCre-mediated recombination was observed in epiblasts before gastrulation. Many genes are transiently expressed in the germ line or at various stages of development^2^ and Cre expression coinciding with such transient expression, even if it is not biologically important, could generate unexpected recombination. In line with a previous report, reanalysis of scRNA-seq datasets and histological analysis during early mouse embryogenesis revealed that the expression of the endogenous Col1α2 gene in epiblasts before gastrulation led to recombination by Col1α2-iCre in epiblasts. Since epiblasts give rise to almost all of the foetal tissues and the extraembryonic mesoderm^22^, these results explained the extensive Cre recombinase activity in various cell types of Col1α2-iCre mice. It is possible that an improved Cre (iCre)^23^ might increase recombination events by Col1α2-iCre, but unexpected Cre recombination can occur in any Cre strain due to Cre expression in undifferentiated cells during the early developmental stage.

Many Cre strains have been used to describe fibroblast behavior. Since fibroblasts originate from multiple cell lineage, genes expressed in fibroblasts might overlap with those in other cell types. Therefore, Cre strains targeting fibroblasts, especially noninducible strains, exhibit unexpected recombinase activity in other cell lineages. Although Col1α2-iCre strain has an extensive recombinase activity, other “classical” fibroblast Cre strains such as *Col1α1-, Pdgfra-, Tcf21-, Ddr2-*Cre also exhibit some degree of unintended recombination in other cell types including intestinal, endothelial, neural cells^6^. There are two ways to improve specificity and accuracy. One is to generate more fibroblast-specific Cre strains, such as ITGA11-Cre, which was reported recently^24^. The other way is to use inducible Cre strains such as the tamoxifen-inducible Cre system or Tet-on/off system. We also analyzed the skin and heart of Col1α2-CreER/mTmG mouse to show relatively specific recombination in cardiac fibroblasts and VSMCs (Fig. 5a,b, Supplementary Fig. S5b,c). However, Col1α2-CreER/mTmG mice had a lower recombination efficiency in cardiac fibroblasts comparing to Col1α2-iCre/mTmG mice (Fig. 5c). Indeed, previous reports indicated that when using an inducible Cre line, the population of recombined cells is much smaller than expected depending on the target gene or cell type of interest^25,26^. Additionally, the inducible Cre system has several limitations: one is that the tissue distribution of exogenous inducers and their active metabolites depends on the administration protocols and target organs^26^; the second is the toxicity of the inducers. For example, high doses of tamoxifen may cause focal cardiac fibrosis in combination with MerCreMer^27,28^. Another is the direct toxicity of temporal Cre expression itself. According to a previous paper, activation of Cre/ERT2 by the administration of tamoxifen caused thymus atrophy, severe anaemia, and abnormal chromosomal rearrangement in haematopoietic cells^28^. Therefore, researchers who plan to use an inducible Cre system should check the recombination efficiency by using a Cre indicator line, like the mTmG system, and evaluate effects of the inducer itself.

Considering the results in the current study, inadvertent and widespread recombination events in various cell types could impact the interpretation of experimental results, and we must be cautious of the results when using Cre-LoxP systems. This study also illustrated a pipeline for the assessment of Cre-mediated recombination events and extensive recombinase activity of Col1α2-iCre. We emphasize the importance of a thorough characterization of the Cre strain, and investigators should take the unexpected Cre recombination events into consideration when they design experimental strategies.

## Methods

### Animals

All animal experiments were performed in accordance with the institutional guidelines of the Institute of Laboratory Animals, Graduate School of Medicine, Kyoto University (Kyoto, Japan), and all experimental protocols were approved by the same institute. Our study was reported in accordance with ARRIVE guidelines.

The noninducible Cre driver line *Col1α2-iCre* is Tg(Col1α2-cre)23Angl, previously reported by Florin et al.^7^, in which the cDNA encoding an improved Cre recombinase (iCre) was fused to the start codon of the Col1α2 gene. The inducible strains *Col1α2-CreER*, a transgenic line, Tg(Col1α2-cre/ERT, and ALPP)7Cpd and the Cre reporter line *(Rosa26R-)mTmG* (B6.129(Cg)-Gt(ROSA)26Sortm4(ACTB-tdTomato, -EGFP)Luo/J) were purchased from the Jackson Laboratory (#029567 and #007676 respectively).

For experiments of adult Col1α2-iCre/mTmG and Col1α2-CreER/mTmG mice, we used 8–10 weeks old mice. For the inducible Cre line, the induction of Cre recombination was started from 7–8 weeks of age by administration of tamoxifen (75 mg/kg body weight) via intraperitoneal injection once every 24 h for a total of 5 consecutive days and samples were collected after a 7‐day waiting period. To obtain Col1α2-iCre/mTmG embryos, three adult mTmG floxed females were housed together with a fertile Col1α2-iCre male overnight and separated the following morning. The vaginal plug was observed, and real-time ultrasound imaging at E5.5 was conducted as previously described^29^ to confirm the pregnancy. For euthanasia, sedation was performed by intraperitoneal administration of a mixture of medetomidine (0.3 mg/kg body weight), midazolam (4 mg/kg body weight) and butorphanol (5 mg/kg body weight).

### Immunostaining

Adult mice were perfused with PBS and 4% paraformaldehyde (PFA). The samples were fixed for 6–24 h in 4% PFA and dehydrated 12 h in sucrose (10% and 30% sucrose in PBS sequentially) before embedding in OCT compound (Sakura Finetek Japan, #45833). Col1α2-iCre/mTmG embryos were collected at E6.5, E9.5, E13.5, and E17.5. Embryos were dissected from the decidua and washed twice with ice-cold PBS. They were fixed for 3 h in 4% PFA and dehydrated in sucrose (10%, 20% and 30% sucrose in PBS sequentially) before embedding in OCT compound. Sections (8 μm) were obtained using a cryostat (Leica Biosystems). For the observation of mT and mG only, samples were washed three times with PBS and mounted with VECTASHIELD HardSet Antifade Mounting Medium with DAPI (Vector Laboratories, #H-1500). For the immunofluorescence study, sections were rinsed with PBS and then incubated in blocking buffer (PBS containing 0.1% Tween 20, 1% bovine serum albumin and 10% normal donkey serum (Jackson ImmunoResearch, #017-000-121) for 1 h at room temperature. Next, they were stained with primary antibodies diluted in blocking buffer overnight at 4 °C. The concentrations of the primary antibodies are provided in Supplementary Table S2. On the following day, the sections were washed three times in ice-cold PBS containing 0.1% Tween20 for 5 min each and incubated with secondary antibodies against the appropriate species at a 1:500 dilution for 1 h at room temperature. They were washed three times in ice-cold PBS for 5 min each. Finally, the samples were mounted with the above-referenced mounting medium. Immunofluorescence studies of cardiac endothelial cells were conducted with DyLight™ 649-conjugated anti-Griffonia simplicifolia isolectin B4 (1:100) (Vector Laboratories, # DL-1208-.5) according to the manufacturer’s instructions.

Fluorescence images were acquired by using BZ-X810 (Keyence) or SP8 Falcon (Leica Biosystems).

### Flow cytometric analysis

Blood samples were collected from the jugular vein using a heparin-containing syringe. Red blood cell lysis was performed with RBC lysis buffer (BioLegend). Then, the samples were washed with FACS buffer (HBSS with 25 mM 4-(2-hydroxyethyl)-1-piperazineethanesulfonic acid (HEPES), 2% FBS, and 2 mM ethylenediaminetetraacetic acid (EDTA)) and resuspended. The cells were blocked with TruStain FcX Plus (BioLegend, #156603) for 5 min at 4 °C.

To prepare a single-cell suspension of noncardiomyocytes, we adopted a previously reported dissociation protocol^30^. Hearts were perfused with ice-cold PBS and removed. The ventricles were finely minced and digested in Hank’s balanced salt solution (HBSS) with collagenase 2 (500 U/ml) (Worthington Biochemical, #LS004176) for 30 min at 37 °C and then incubated in HBSS with collagenase/dispase (1 mg/mL) (Merck, #11097113001) for 20 min at 37 °C. To deactivate the enzymes, the samples were washed with ice-cold HBSS and passed through a 40 μm cell strainer (Corning, #352340). After red blood cell lysis, the cardiac samples were washed with FACS buffer and resuspended and then blocked with TruStain FcX Plus for 5 min at 4 °C.

Bone marrow cells were flushed from femurs and tibias with RPMI supplemented with 25 mM HEPES and 10% FBS and filtered through a 40 μm cell strainer (Falcon, #352340). After the red blood cells were lysed, the samples were washed with 2% FBS-containing HBSS buffer and resuspended.

The cells were stained with conjugated primary antibodies (see Supplementary Table S1) and then analysed by flow cytometry with FACSAria™ IIu (BD Biosciences) and FACSDiva software (BD Biosciences) or FlowJo software (Tree Star).

### Bone marrow transplantation

Bone marrow cells were collected from Col1α2-iCre/mTmG mice and injected 5.0 ´ 10^6^ cells into wild-type recipient mice that received a total radiation dose of 12 Gy in two 6 Gy fractions separated by a 3-h interval (Supplementary Fig. S2b).

### scRNA-seq data

Transcriptional data at E4.5, E5.25, E5.5, E6.25 and E6.5 were downloaded in raw-count forms from the Gene Expression Omnibus of the National Center for Biotechnology Information GSE109071 dataset. The data were processed with the R (4.1.3) package Seurat version 4 by weighted-nearest neighbour analysis^31^.

The other datasets spanning E6.75 to E13.5 were obtained in processed forms from http://tome.gs.washington.edu/^20^. These objects were analysed with R (4.1.3) package Seurat version 4.

### Statistics

All results are presented as the mean ± SEM. Differences between two groups were compared by Student’s t test as a parametric comparison test or by Wilcoxon rank sum test as a nonparametric comparison test. The analysis and plots were generated using the R (4.1.3) package ggplot2 version 3.

### Supplementary Information

Supplementary Information.

## Data Availability

All data generated or analyzed during this study are included in this published article and its supplementary information file.
