# Supplementary material for: Col1α2-Cre-mediated recombination occurs in various cell types due to Cre expression in epiblasts
Source: Sci Rep. 2023 Dec 18;13:22483. doi: 10.1038/s41598-023-50053-z (PMC10728165; doi:10.1038/s41598-023-50053-z)
Supplement: Supplementary file 1 — Supplementary Information. [file 41598_2023_50053_MOESM1_ESM.pdf]

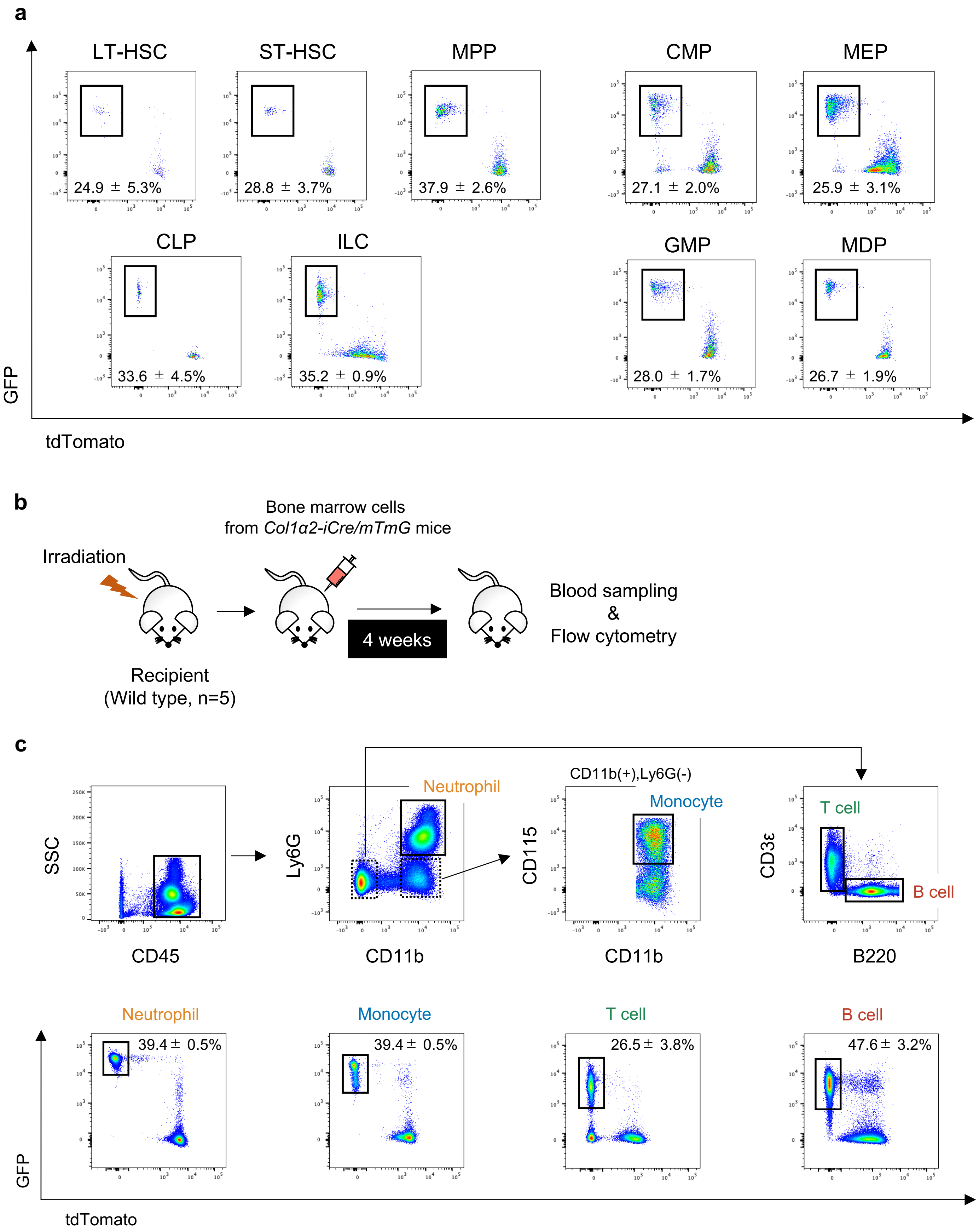

Supplementary Figure S1. (a) GFP expression in each cell type in the bone marrow of Col1α2-iCre/mTmG mice. (b) Schematic summary of bone marrow transplantation. (c) Flow cytometry of the peripheral blood of recipient mice after bone marrow transplantation.

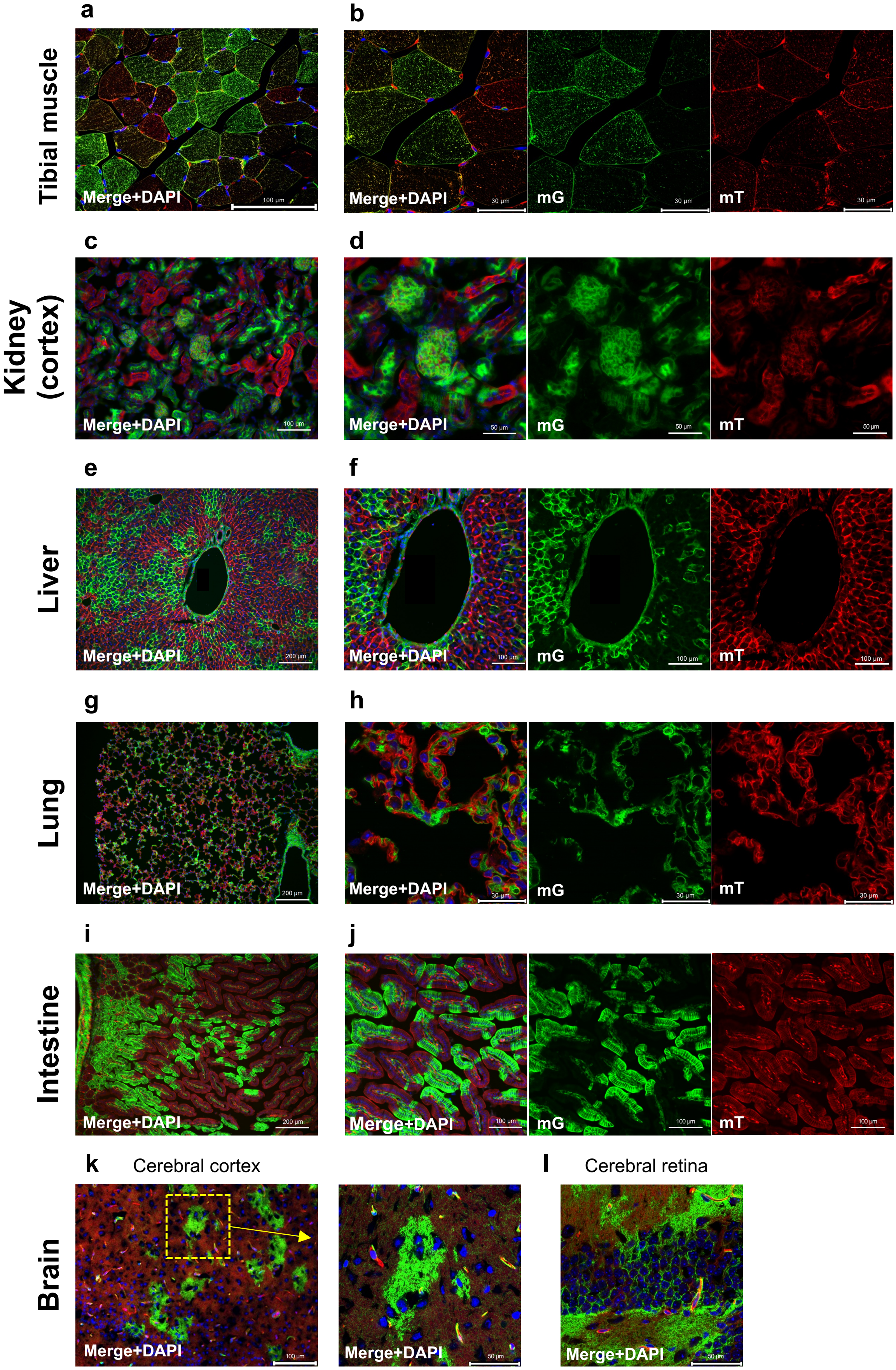

Supplementary Figure S2. Various organs of Col1α2-iCre/mTmG mouse. (a) & (b) Tibial muscles (Scale bars: 100 μm & 30 μm each). (c) & (d) Kidney (Scale bars: 100 μm & 50 μm each). (e) & (f) Liver (Scale bars: 200 μm & 10 μm each). (g) & (h) Lung (Scale bars: 200 μm & 30 μm each). (i) & (j) Intestine (Scale bars: 200 μm & 10 μm each). (k) Cerebral cortex (Scale bar: 100 μm & 50 μm each). (l) Cerebral retina (Scale bar: 50 μm).

a

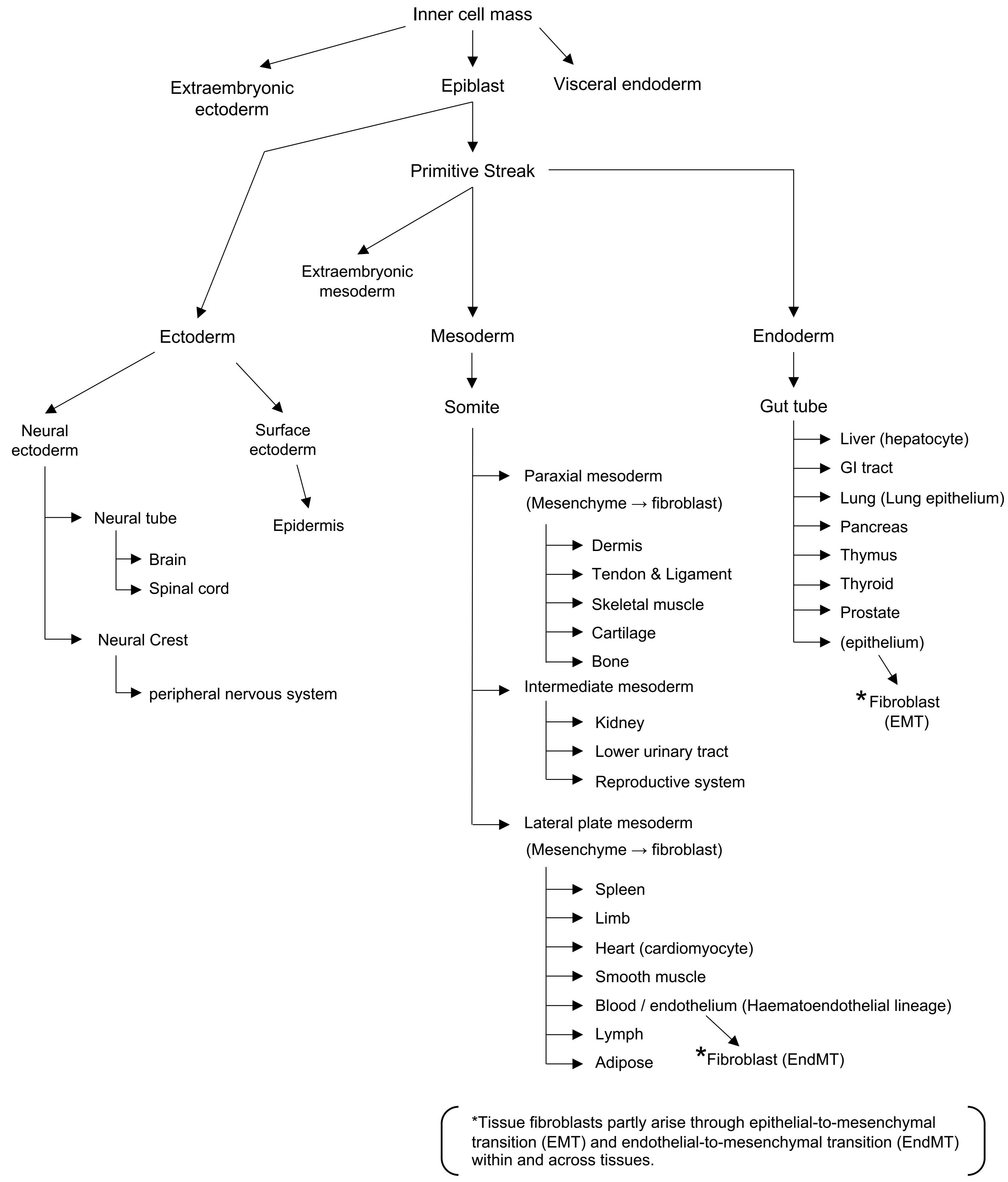

Supplementary Figure S3. (a) Organ or tissue developmental tree during embryogenesis.

b

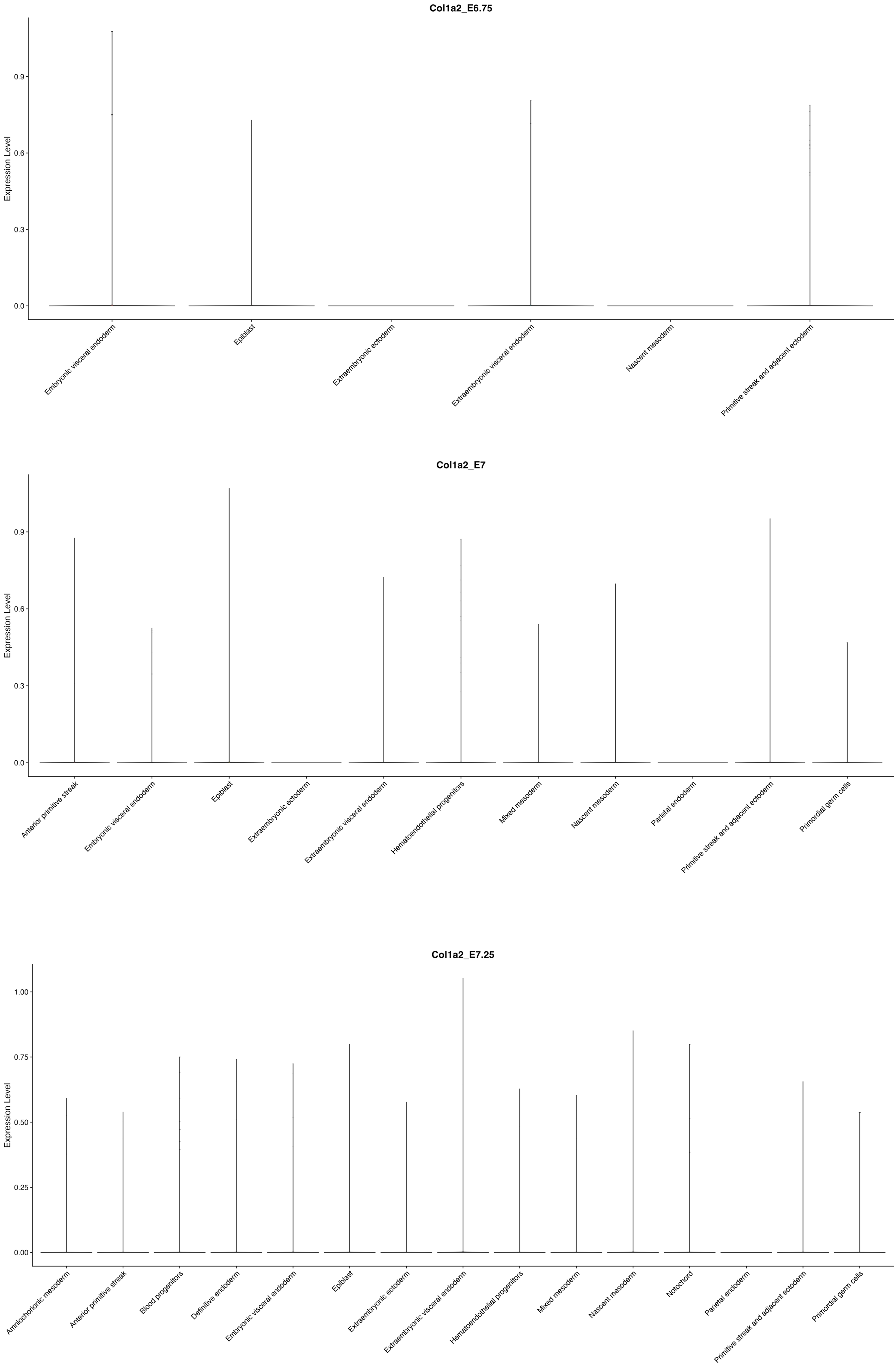
Col1a2_E6.75
Expression Level
0.0
0.3
0.6
0.9
Embryonic visceral endoderm
Epiblast
Extraembryonic ectoderm
Extraembryonic visceral endoderm
Nascent mesoderm
Primitive streak and adjacent ectoderm
Col1a2_E7
Expression Level
0.0
0.3
0.6
0.9
Anterior primitive streak
Embryonic visceral endoderm
Epiblast
Extraembryonic ectoderm
Extraembryonic visceral endoderm
Hematoendothelial progenitors
Mixed mesoderm
Nascent mesoderm
Parietal endoderm
Primitive streak and adjacent ectoderm
Primordial germ cells
Col1a2_E7.25
Expression Level
0.00
0.25
0.50
0.75
1.00
Amniochorionic mesoderm
Anterior primitive streak
Blood progenitors
Definitive endoderm
Embryonic visceral endoderm
Epiblast
Extraembryonic ectoderm
Extraembryonic visceral endoderm
Hematoendothelial progenitors
Mixed mesoderm
Nascent mesoderm
Notochord
Parietal endoderm
Primitive streak and adjacent ectoderm
Primordial germ cells

c

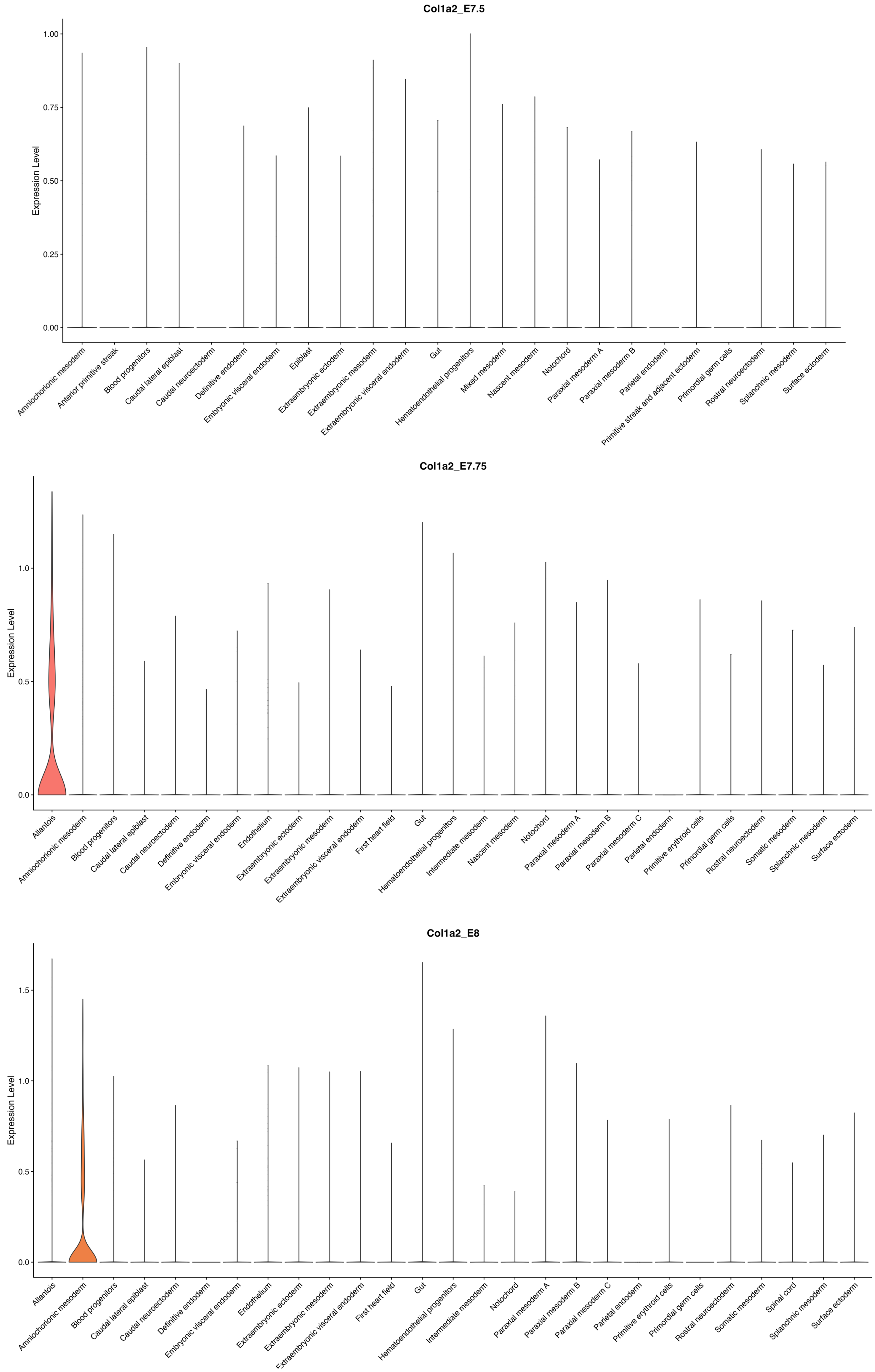

Col1a2_E7.5
Expression Level
Amniochorionic mesoderm
Anterior primitive streak
Blood progenitors
Caudal lateral epiblast
Caudal neuroectoderm
Definitive endoderm
Embryonic visceral endoderm
Epiblast
Extraembryonic ectoderm
Extraembryonic mesoderm
Extraembryonic visceral endoderm
Gut
Hematoendothelial progenitors
Mixed mesoderm
Nascent mesoderm
Notochord
Paraxial mesoderm A
Paraxial mesoderm B
Parietal endoderm
Primitive streak and adjacent ectoderm
Primordial germ cells
Rostral neuroectoderm
Splanchnic mesoderm
Surface ectoderm
Col1a2_E7.75
Allantois
First heart field
Endothelium
Intermediate mesoderm
Paraxial mesoderm C
Primitive erythroid cells
Somatic mesoderm
Col1a2_E8
Spinal cord

d

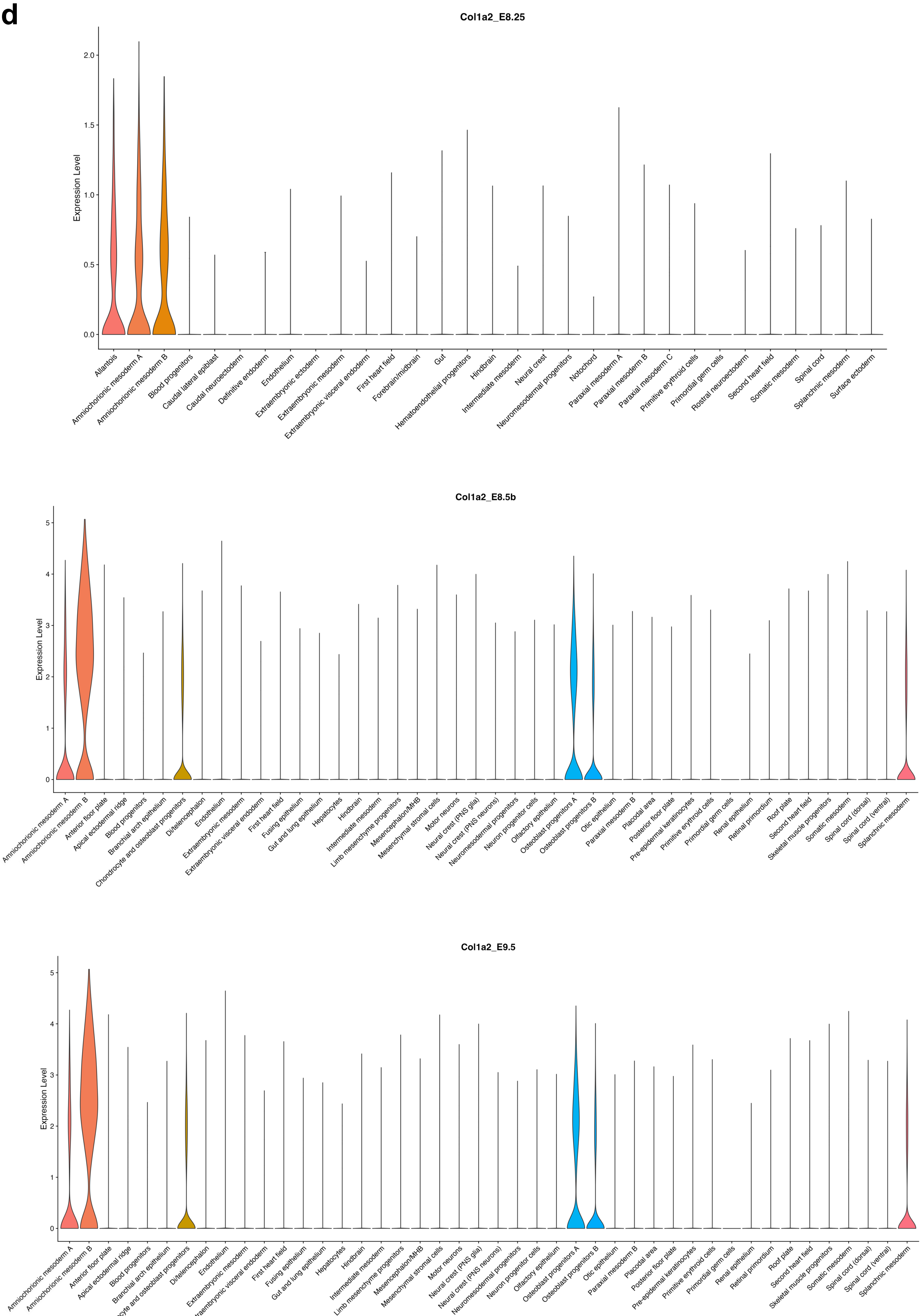
Col1a2_E8.25
Expression Level
0.0
0.5
1.0
1.5
2.0
Allantois
Amniochorionic mesoderm A
Amniochorionic mesoderm B
Blood progenitors
Caudal lateral epiblast
Caudal neuroectoderm
Definitive endoderm
Endothelium
Extraembryonic ectoderm
Extraembryonic mesoderm
Extraembryonic visceral endoderm
First heart field
Forebrain/midbrain
Gut
Hematoendothelial progenitors
Hindbrain
Intermediate mesoderm
Neural crest
Neuromesodermal progenitors
Notochord
Paraxial mesoderm A
Paraxial mesoderm B
Paraxial mesoderm C
Primitive erythroid cells
Primordial germ cells
Rostral neuroectoderm
Second heart field
Somatic mesoderm
Spinal cord
Splanchnic mesoderm
Surface ectoderm
Col1a2_E8.5b
Expression Level
0
1
2
3
4
5
Amniochorionic mesoderm A
Amniochorionic mesoderm B
Anterior floor plate
Apical ectodermal ridge
Blood progenitors
Branchial arch epithelium
Chondrocyte and osteoblast progenitors
Diencephalon
Endothelium
Extraembryonic mesoderm
Extraembryonic visceral endoderm
First heart field
Fusing epithelium
Gut and lung epithelium
Hepatocytes
Hindbrain
Intermediate mesoderm
Limb mesenchyme progenitors
Mesencephalon/MHB
Mesenchymal stromal cells
Motor neurons
Neural crest (PNS glia)
Neural crest (PNS neurons)
Neuromesodermal progenitors
Neuron progenitor cells
Olfactory epithelium
Osteoblast progenitors A
Osteoblast progenitors B
Otic epithelium
Paraxial mesoderm B
Placodal area
Posterior floor plate
Pre-epidermal keratinocytes
Primitive erythroid cells
Primordial germ cells
Renal epithelium
Retinal primordium
Roof plate
Second heart field
Skeletal muscle progenitors
Somatic mesoderm
Spinal cord (dorsal)
Spinal cord (ventral)
Splanchnic mesoderm
Col1a2_E9.5
Expression Level
0
1
2
3
4
5
Amniochorionic mesoderm A
Amniochorionic mesoderm B
Anterior floor plate
Apical ectodermal ridge
Blood progenitors
Branchial arch epithelium
Chondrocyte and osteoblast progenitors
Diencephalon
Endothelium
Extraembryonic mesoderm
Extraembryonic visceral endoderm
First heart field
Fusing epithelium
Gut and lung epithelium
Hepatocytes
Hindbrain
Intermediate mesoderm
Limb mesenchyme progenitors
Mesencephalon/MHB
Mesenchymal stromal cells
Motor neurons
Neural crest (PNS glia)
Neural crest (PNS neurons)
Neuromesodermal progenitors
Neuron progenitor cells
Olfactory epithelium
Osteoblast progenitors A
Osteoblast progenitors B
Otic epithelium
Paraxial mesoderm B
Placodal area
Posterior floor plate
Pre-epidermal keratinocytes
Primitive erythroid cells
Primordial germ cells
Renal epithelium
Retinal primordium
Roof plate
Second heart field
Skeletal muscle progenitors
Somatic mesoderm
Spinal cord (dorsal)
Spinal cord (ventral)
Splanchnic mesoderm

e

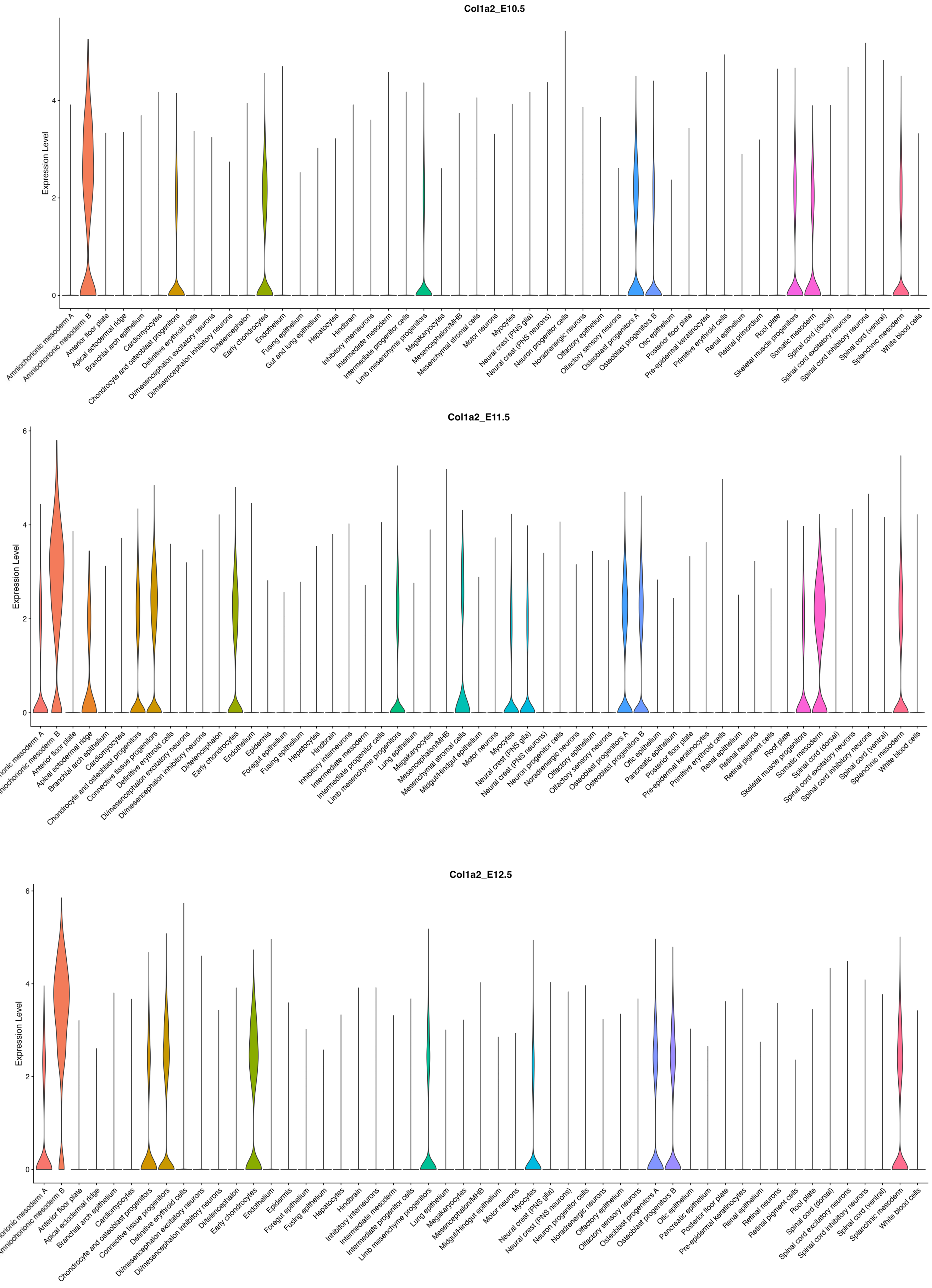

Col1a2_E10.5
Expression Level
0
2
4
Amniochorionic mesoderm A
Amniochorionic mesoderm B
Anterior floor plate
Apical ectodermal ridge
Branchial arch epithelium
Cardiomyocytes
Chondrocyte and osteoblast progenitors
Definitive erythroid cells
Di/mesencephalon excitatory neurons
Di/mesencephalon inhibitory neurons
Di/telencephalon
Early chondrocytes
Endothelium
Fusing epithelium
Gut and lung epithelium
Hepatocytes
Hindbrain
Inhibitory interneurons
Intermediate mesoderm
Intermediate progenitor cells
Limb mesenchyme progenitors
Megakaryocytes
Mesencephalon/MHB
Mesenchymal stromal cells
Motor neurons
Myocytes
Neural crest (PNS glia)
Neural crest (PNS neurons)
Neuron progenitor cells
Noradrenergic neurons
Olfactory epithelium
Olfactory sensory neurons
Osteoblast progenitors A
Osteoblast progenitors B
Otic epithelium
Posterior floor plate
Pre-epidermal keratinocytes
Primitive erythroid cells
Renal epithelium
Retinal primordium
Roof plate
Skeletal muscle progenitors
Somatic mesoderm
Spinal cord (dorsal)
Spinal cord excitatory neurons
Spinal cord inhibitory neurons
Spinal cord (ventral)
Splanchnic mesoderm
White blood cells
Col1a2_E11.5
Expression Level
0
2
4
6
Amniochorionic mesoderm A
Amniochorionic mesoderm B
Anterior floor plate
Apical ectodermal ridge
Branchial arch epithelium
Cardiomyocytes
Chondrocyte and osteoblast progenitors
Connective tissue progenitors
Definitive erythroid cells
Di/mesencephalon excitatory neurons
Di/mesencephalon inhibitory neurons
Di/telencephalon
Early chondrocytes
Endothelium
Epidermis
Foregut epithelium
Fusing epithelium
Hepatocytes
Hindbrain
Inhibitory interneurons
Intermediate mesoderm
Intermediate progenitor cells
Limb mesenchyme progenitors
Lung epithelium
Megakaryocytes
Mesencephalon/MHB
Mesenchymal stromal cells
Midgut/Hindgut epithelium
Motor neurons
Myocytes
Neural crest (PNS glia)
Neural crest (PNS neurons)
Neuron progenitor cells
Noradrenergic neurons
Olfactory epithelium
Olfactory sensory neurons
Osteoblast progenitors A
Osteoblast progenitors B
Otic epithelium
Pancreatic epithelium
Posterior floor plate
Pre-epidermal keratinocytes
Primitive erythroid cells
Renal epithelium
Retinal neurons
Retinal pigment cells
Roof plate
Skeletal muscle progenitors
Somatic mesoderm
Spinal cord (dorsal)
Spinal cord excitatory neurons
Spinal cord inhibitory neurons
Spinal cord (ventral)
Splanchnic mesoderm
White blood cells
Col1a2_E12.5
Expression Level
0
2
4
6
Amniochorionic mesoderm A
Amniochorionic mesoderm B
Anterior floor plate
Apical ectodermal ridge
Branchial arch epithelium
Cardiomyocytes
Chondrocyte and osteoblast progenitors
Connective tissue progenitors
Definitive erythroid cells
Di/mesencephalon excitatory neurons
Di/mesencephalon inhibitory neurons
Di/telencephalon
Early chondrocytes
Endothelium
Epidermis
Foregut epithelium
Fusing epithelium
Hepatocytes
Hindbrain
Inhibitory interneurons
Intermediate mesoderm
Intermediate progenitor cells
Limb mesenchyme progenitors
Lung epithelium
Megakaryocytes
Mesencephalon/MHB
Midgut/Hindgut epithelium
Motor neurons
Myocytes
Neural crest (PNS glia)
Neural crest (PNS neurons)
Neuron progenitor cells
Noradrenergic neurons
Olfactory epithelium
Olfactory sensory neurons
Osteoblast progenitors A
Osteoblast progenitors B
Otic epithelium
Pancreatic epithelium
Posterior floor plate
Pre-epidermal keratinocytes
Renal epithelium
Retinal neurons
Retinal pigment cells
Roof plate
Spinal cord (dorsal)
Spinal cord excitatory neurons
Spinal cord inhibitory neurons
Spinal cord (ventral)
Splanchnic mesoderm
White blood cells

f

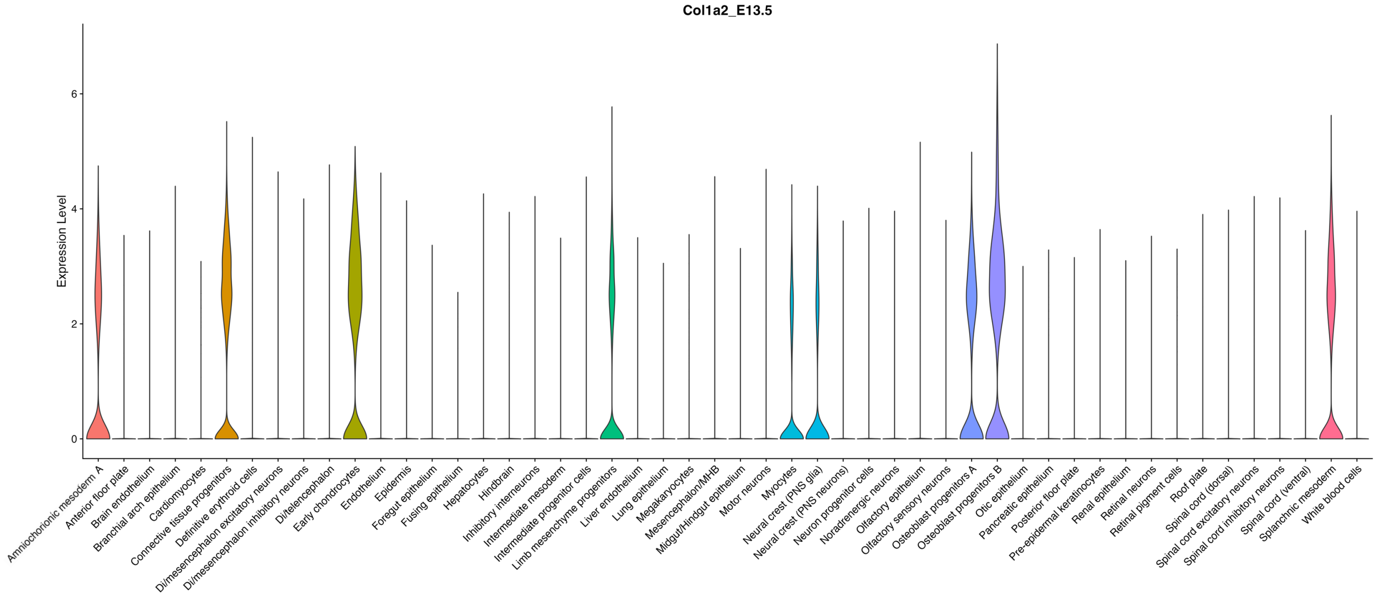

Supplementary Figure S3. Violin plot of *Col1α2* expression at (b) E6.75, E7, E7.25, (c) E7.5, E7.75, E8, (d) E8.25, E8.5, E9.5, (e) E10.5, E11.5, E12.5, (f) E13.5.

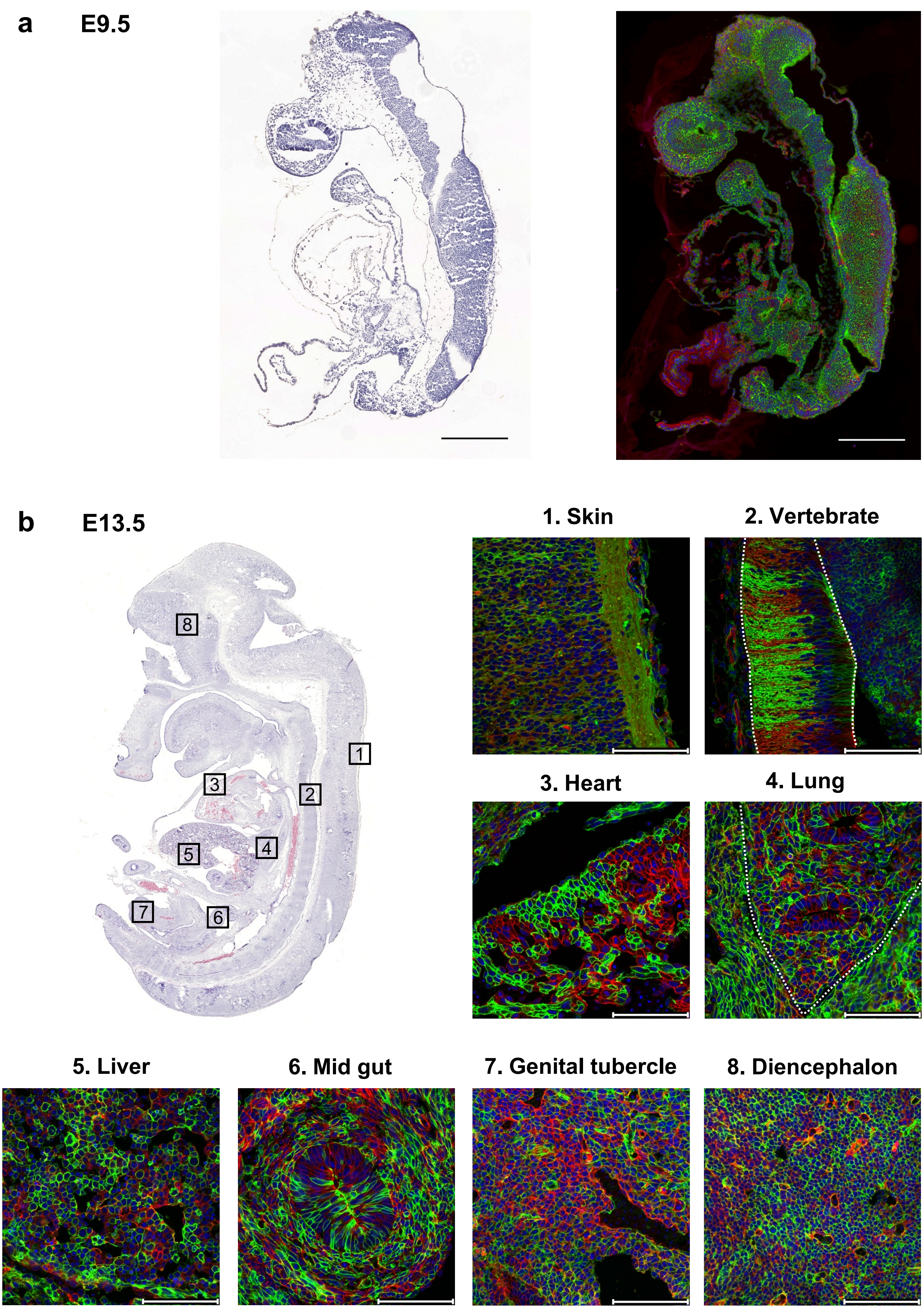

Supplementary Figure S4. Embryo of Col1α2-iCre/mTmG mice. (a) whole image of embryo at E9.5, HE stain and Immunofluorescence image. Scale bar: 300 μm. (b) whole image of embryo at E13.5 (HE stain), Immunofluorescence image of skin, vertebrate (between the dotted lines), myocardium, lung (inside of the dotted line), liver, mid gut, genital tubercle and diencephalon. Scale bar: 50 μm.

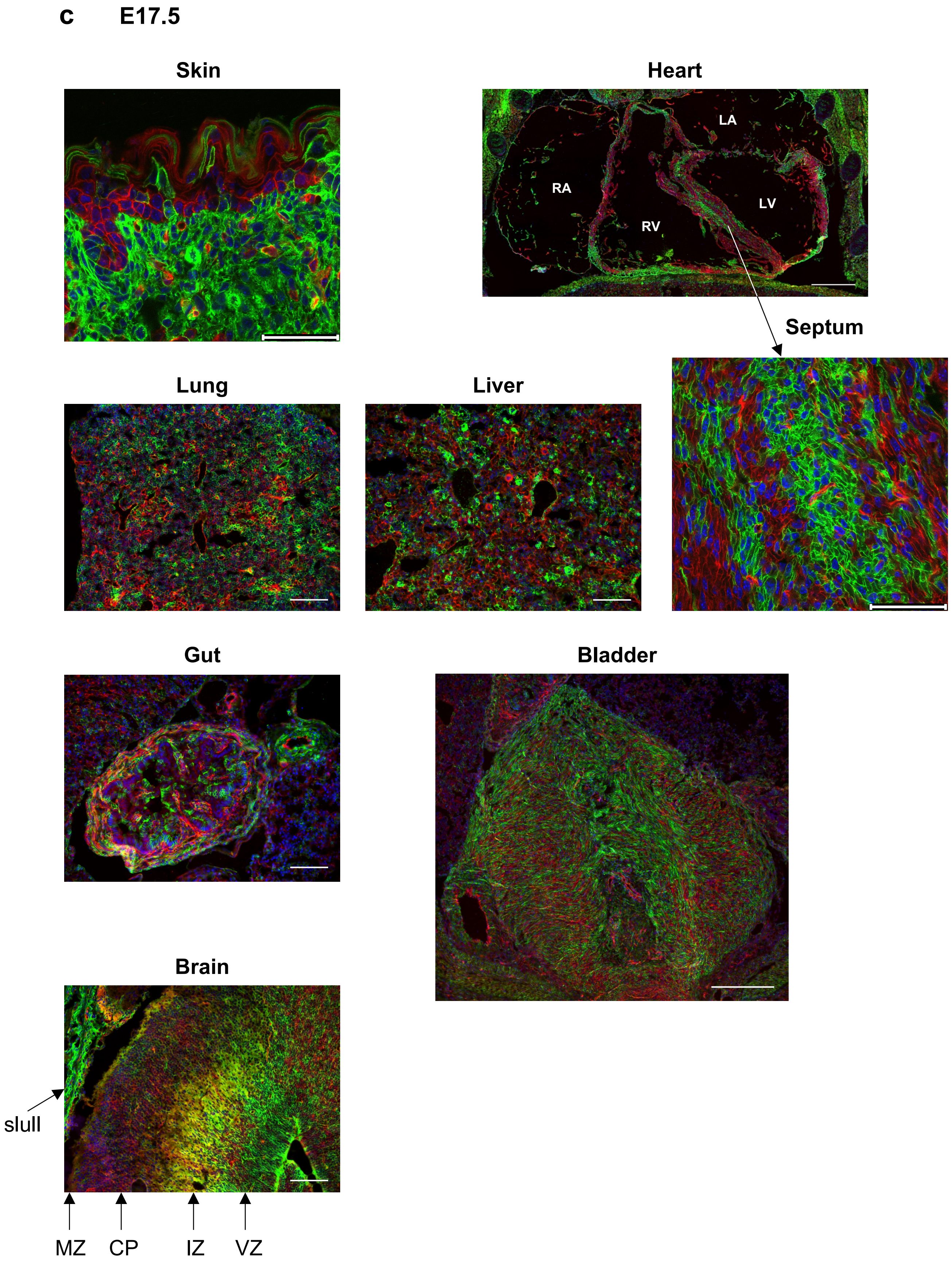

Supplementary Figure S4. Embryo of Col1α2-iCre/mTmG mice. (c) Immunofluorescence image at E17.5 of skin, heart (whole image and septum), lung, liver, gut, liver, bladder and brain. MZ: marginal zone, CP: cortical plate, IZ: intermediate zone, VZ: ventricular zone. Scale bars: 500 μm (whole image of heart), 300 μm (bladder), 50 μm (skin, septum), 100 μm (the others).

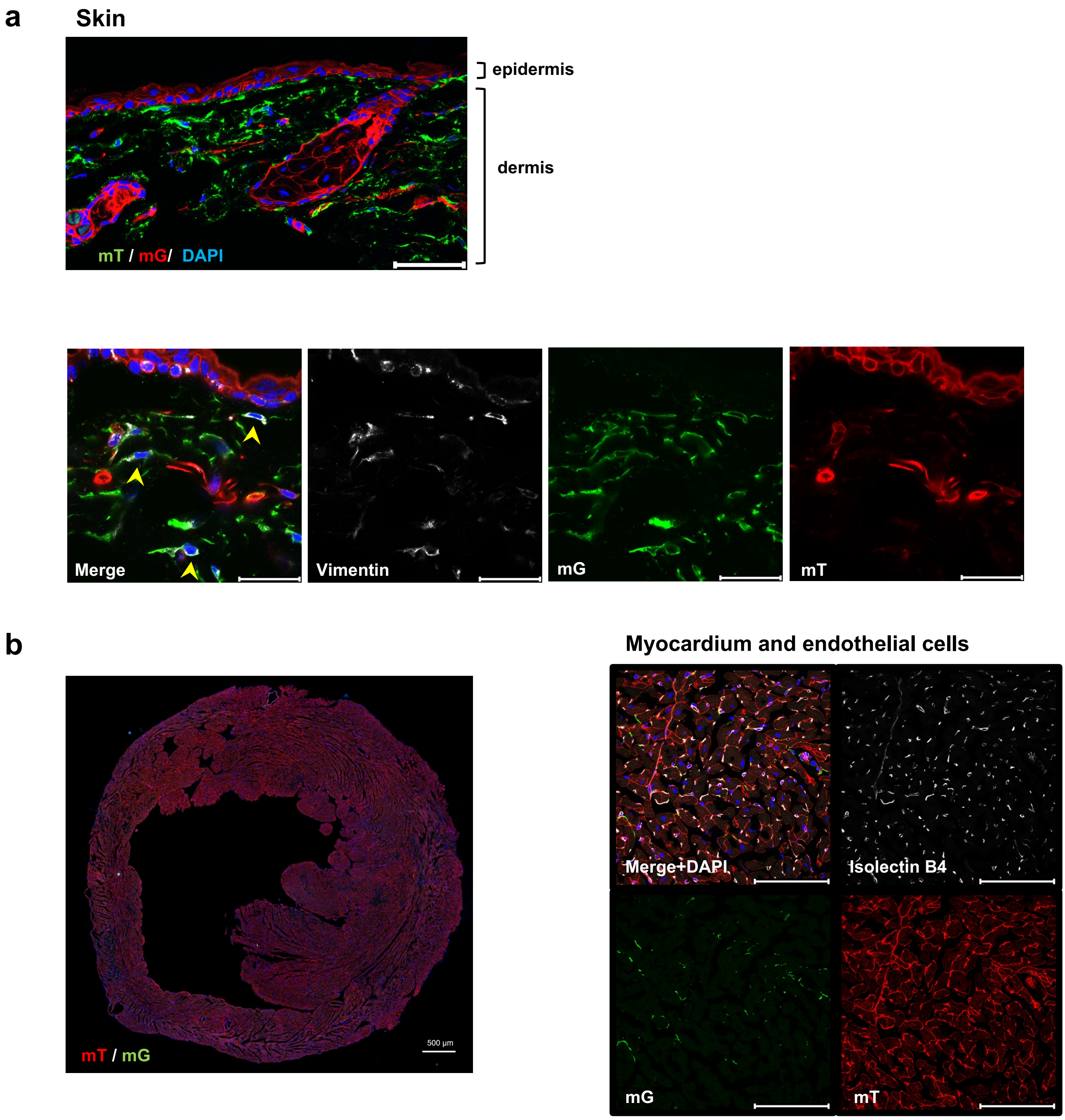

Supplementary Figure S5. Cre-mediated recombination of Col1α2-CreER/mTmG skin and heart.

(a) The upper is dermis and epidermis and the lower is Immunohistochemistry of dermal fibroblasts. Arrowheads indicate examples of both vimentin and GFP positive cells. Scale bars: 500 μm and 30 μm.

(b) Left: whole image of Col1α2-CreER/mTmG heart. Right: magnified image in which endothelial cells were stained by isolectin B4 (Scale bar: 30 μm). GFP positive cardiomyocyte and endothelial cell was not detected.

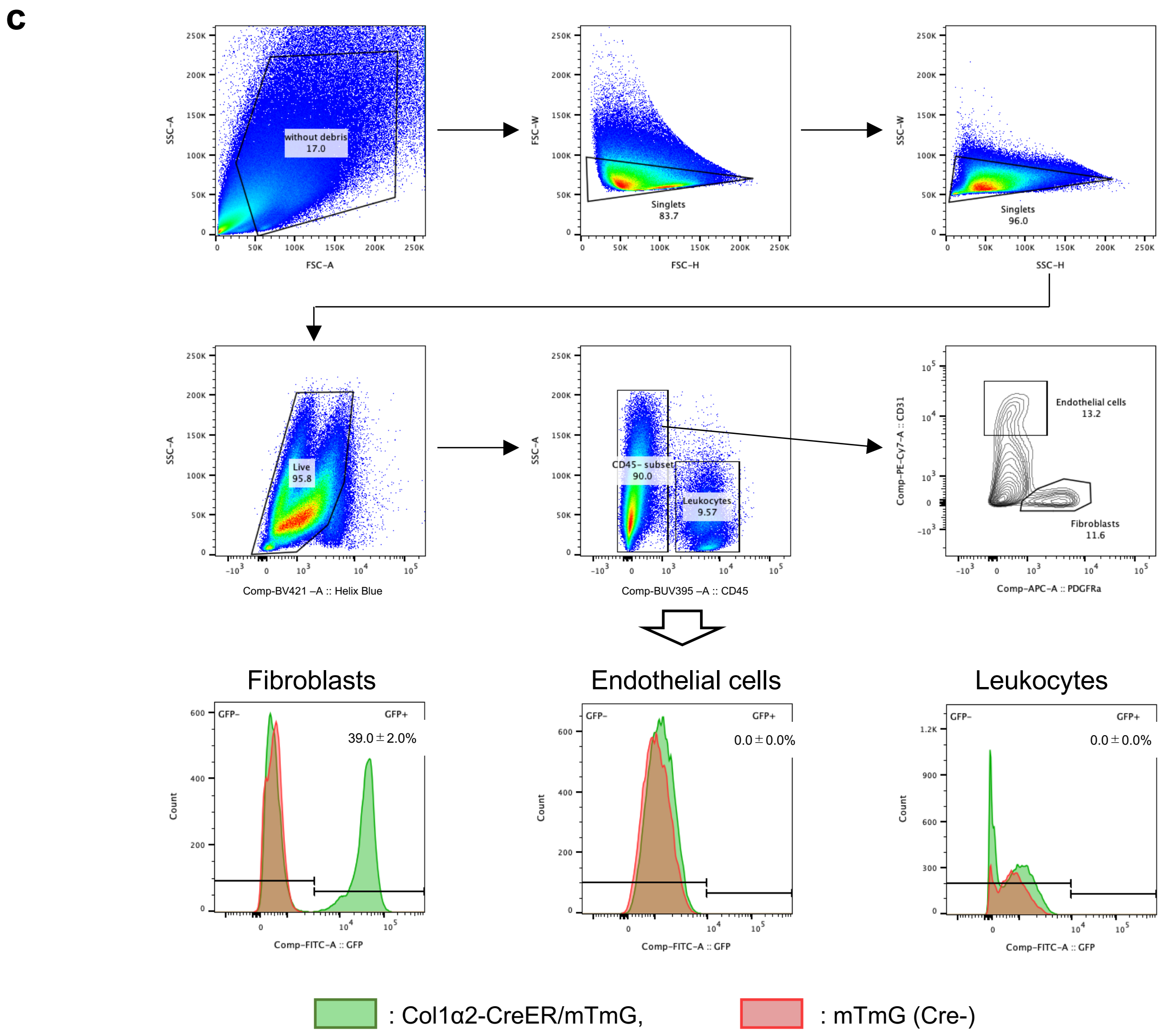

Supplementary Figure S5. (c) Flow cytometry of the non-myocytes of Col1α2-CreER/mTmG hearts and GFP expression in each cell type (n=3).

Supplementary Table S1: Summary of flow cytometric analysis

■ Peripheral blood cells of Col1α2-iCre/mTmG mice (n=4)

cell type	type-specific marker	%GFP positive cells
Neutrophil	CD45+, CD11b+, Ly6G+	22.5 ± 5.2%
Monocyte	CD45+, CD11b+, Ly6G-, CD115+	25.4 ± 3.5%
T cell	CD45+, CD11b-, CD3ε+	37.0 ± 3.6%
B cell	CD45+, CD11b-, B220+	28.9 ± 2.5%

■ Bone marrow cells of Col1α2-iCre/mTmG mice (n=3)

cell type	type-specific marker	%GFP positive cells
LT-HSC	Lineage-,Sca-1+,c-Kit+,CD150+,CD48-	24.9 ± 5.3%
ST-HSC	Lineage-,Sca-1+,c-Kit+,CD150-,CD48-	28.8 ± 3.7%
MPP	Lineage-,Sca-1+,c-Kit+,CD150-,CD48+	37.9 ± 2.6%
CMP	Lineage-,Sca-1-,c-Kit+,CD34+,CD16/32-	27.1 ± 2.0%
GMP	Lineage-,Sca-1-,c-Kit+,CD34+,CD16/32+,CD115-	28.0 ± 1.7%
MDP	Lineage-,Sca-1-,c-Kit+,CD34+,CD16/32+,CD115+	26.7 ± 1.9%
MEP	Lineage-,Sca-1-,c-Kit+,CD34-,CD16/32-	25.9 ± 3.1%
CLP	Lineage-,Sca-1 low,c-Kit low,CD135+,CD127+	33.6 ± 4.5%
ILC	Lineage-,Sca-1+,c-Kit-	35.2 ± 0.9%

■ Cardiac cells of Col1α2-iCre/mTmG mice

cell type	type-specific marker	%GFP positive cells
Fibroblast (n=5)	CD45-,CD31-,PDGFRα+	72.4 ± 4.5%
Endothelial cell (n=5)	CD45-,CD31+	22.6 ± 7.1%
Macrophage (n=3)	CD45+,CD11b+,CD64+,Ly6C-	26.8 ± 4.1%
T cell (n=3)	CD45+, CD11b-, CD3ε+	32.1 ± 4.2%
B cell (n=3)	CD45+, CD11b-, B220+	25.2 ± 1.6%

■ Percentages of GFP-positive populations of Col1α2-iCre/mTmG and Col1α2-CreER/mTmG hearts

	Col1α2-iCre/mTmG	Col1α2-CreER/mTmG	*P* value
Fibroblast (CD45-,CD31-,PDGFRα+)	73.4 ± 4.8%	39.0 ± 2.0%	< 0.001
Endothelial cell (CD45-,CD31+)	22.6 ± 7.1%	0.0 ± 0.0%	0.04
Leukocyte (CD45+)	41.5 ± 8.7%	0.0 ± 0.0%	0.01

Supplementary Table S2: Reagent and resource

■ Flow cytometry

Antigen	Clone	Fluorescent dye	Company	Identifier
CD31	390	PE-Cyanine7	BioLegend	102418
CD45	30-F11	BUV395	BD Biosciences	564279
PDGFRa	APA5	APC	BioLegend	135908
CD11b	M1/70	BUV737	BD Biosciences	612800
Ly6G	1A8	BV605	BioLegend	127639
CD64	X54-5/7.1	PE-Cyanine7	BioLegend	139314
Ly6C	HK1.4	APC-Cyanine7	BioLegend	128025
CD3e	145-2C11	APC	BioLegend	100311
B220	RA3-6B2	BV421	BioLegend	103239
CD115	AFS98	PE-Cyanine7	BioLegend	135523
CD115	AFS98	BUV395	BD Biosciences	750886
Lineage cockatil		BV421	BioLegend	133311
c-Kit	2B8	APC-Cyanine7	BioLegend	105825
Sca1	D7	BV605	BioLegend	108133
CD48	HM48-1	APC	BioLegend	103411
CD150	TC15-12F12.2	PE-Cyanine7	BioLegend	115913
CD34	RAM34	eFluor 660	ThermoFisher	50-0341-82
CD16/32	93	PE-Cyanine7	BioLegend	101317
CD135	A2F10	APC	BioLegend	135309
CD127	A7R34	PE-Cyanine7	BioLegend	135013
7-AAD			BioLegend	420404
Helix NP™ Blue			BioLegend	25305

■ Immunohistochemistry

Antigen		Host	Company	Identifier
Vimentin	Monoclonal	Rabbit	Cell Signaling Technology	5741S
αSMA	Polyclonal	Goat	Novus Biologicals	NB300-978
Isolectin B4			Vector Laboratories	DL-1208-.5
CD68	Monoclonal	Rat	Bio-Rad Laboratories	MCA1957GA
